# Supplementary material for: A high-resolution mRNA expression time course of embryonic development in zebrafish
Source: eLife. 2017 Nov 16;6:e30860. doi: 10.7554/eLife.30860 (PMC5690287; doi:10.7554/eLife.30860)
Supplement: Supplementary file 6. [file elife-30860-supp6.zip › biolayout-clusters-files/Cluster171-genes.html]

Cluster171


# Cluster171: Genes

| Ensembl ID | Gene Name | Chr | Start | End | Biotype |
| --- | --- | --- | --- | --- | --- |
| ENSDARG00000101697 | VWDE | 15 | 34136596 | 34198754 | protein\_coding |
| ENSDARG00000055715 | capn8 | 22 | 26380642 | 26612706 | protein\_coding |
| ENSDARG00000068191 | cdh15 | 7 | 55797111 | 55844260 | protein\_coding |
| ENSDARG00000074677 | frem3 | 1 | 34882062 | 34921533 | protein\_coding |
| ENSDARG00000039052 | klhl40a | 2 | 21668895 | 21677069 | protein\_coding |
| ENSDARG00000057317 | nexn | 8 | 18481863 | 18502176 | protein\_coding |
| ENSDARG00000078696 | synpo2lb | 12 | 26318066 | 26339147 | protein\_coding |
| ENSDARG00000020594 | txlnba | 20 | 36942908 | 36984386 | protein\_coding |
